# TAK1 regulates caspase 8 activation and necroptotic signaling via multiple cell death checkpoints

**DOI:** 10.1038/cddis.2016.294

**Published:** 2016-09-29

**Authors:** Xiaoyun Guo, Haifeng Yin, Yi Chen, Lei Li, Jing Li, Qinghang Liu

**Affiliations:** 1 Department of Physiology and Biophysics, University of Washington, Seattle 98195, WA, USA

## Abstract

Necroptosis has emerged as a new form of programmed cell death implicated in a number of pathological conditions such as ischemic injury, neurodegenerative disease, and viral infection. Recent studies indicate that TGF*β*-activated kinase 1 (TAK1) is nodal regulator of necroptotic cell death, although the underlying molecular regulatory mechanisms are not well defined. Here we reported that TAK1 regulates necroptotic signaling as well as caspase 8-mediated apoptotic signaling through both NF*κ*B-dependent and -independent mechanisms. Inhibition of TAK1 promoted TNF*α*-induced cell death through the induction of RIP1 phosphorylation/activation and necrosome formation. Further, inhibition of TAK1 triggered two caspase 8 activation pathways through the induction of RIP1-FADD-caspase 8 complex as well as FLIP cleavage/degradation. Mechanistically, our data uncovered an essential role for the adaptor protein TNF receptor-associated protein with death domain (TRADD) in caspase 8 activation and necrosome formation triggered by TAK1 inhibition. Moreover, ablation of the deubiqutinase CYLD prevented both apoptotic and necroptotic signaling induced by TAK1 inhibition. Finally, blocking the ubiquitin-proteasome pathway prevented the degradation of key pro-survival signaling proteins and necrosome formation. Thus, we identified new regulatory mechanisms underlying the critical role of TAK1 in cell survival through regulation of multiple cell death checkpoints. Targeting key components of the necroptotic pathway (e.g., TRADD and CYLD) and the ubiquitin-proteasome pathway may represent novel therapeutic strategies for pathological conditions driven by necroptosis.

Apoptosis and necrosis are two morphologically and mechanistically distinct forms of cell death. Apoptosis is induced by death receptor- or mitochondria-mediated pathways, showing distinct morphological features including membrane blebing, cell shrinkage, nuclear fragmentation, and chromatin condensation.^1^ Recent studies indicate that certain forms of necrosis occur in a highly regulated, programmed fashion. The programmed necrosis, also termed necroptosis, has been described as a caspase-independent cell death that can occur in ATP- or mitochondria-depleted settings, characterized by plasma membrane disruption, organelle swelling, cell lysis, and inflammation.^2, 3, 4, 5^ Apoptosis and necroptosis are induced via specific dearth receptors such as tumor necrosis factor receptor 1 (TNFR1), among other modules.

The pleiotropic nature of TNFR1 signaling results from the formation of different signaling complexes.^6, 7, 8^ Under normal conditions, ligation of TNFR1 induces the assembly of a plasma membrane bound signaling complex, termed complex I, which contains TNF receptor-associated protein with death domain (TRADD), TNF receptor-associated protein 2 (TRAF2), receptor-interacting protein 1 (RIP1), and cellular inhibitor of apoptosis protein 1 and 2 (cIAP1 and cIAP2).^8^ The recruitment of TGF*β*-activated kinase 1 (TAK1) and the I*κ*B kinase (IKK) complex to the complex I leads to the activation of NF*κ*B, which drives the transcription of pro-survival genes. Under certain conditions such as inhibition of NF*κ*B signaling or protein synthesis, the TNFR1 complex then internalizes and converts to a cell death-inducing complex, termed complex II, with additional recruitment of Fas-associated protein with death domain (FADD) and caspase 8.^8, 9, 10^ TNFR1 signaling can also induce the formation of another cell death-inducing complex, known as necrosome, consisting of RIP1, RIP3, and FADD.^11^

The kinase activity of RIP1 is essential for RIP1–RIP3 interaction and necroptosis,^3, 4, 5, 12^ but not essential for TNFR1-mediated NF*κ*B activation.^13^ RIP3 depletion has been regarded as a gold standard to differentiate necroptosis from other forms of cell death,^14^ but recent studies indicated it may also alter apoptotic signaling.^15, 16^ In addition to phosphorylation, unbiquitination of RIP1 has been suggested to be another important regulatory mechanism in TNFR1 signaling. TRAF2 and cIAP1/2 act as ubiquitin ligases for RIP1, and lysine 63-linked ubiquitination of RIP1 on lysine 377 is believed to prevent the formation of complex II by stabilizing complex I.^17, 18^ On the other hand, the deubiquitinating enzyme cylindromatosis (CYLD) promotes necroptotic signaling by augmenting RIP1–RIP3 interaction.^19^

TAK1 (gene name *Map3k7*), a member of the mitogen-activated kinase kinase kinase (Map3k) family,^20^ has recently emerged as nodal regulator of necroptotic signaling, although the underlying molecular regulatory pathways remain poorly defined.^16, 21, 22, 23^ *In vivo* studies showed that tissue-specific ablation of TAK1 led to spontaneous cell death, inflammation, and fibrosis in various organs, and these effects were largely reversed by TNFR1 deletion, further supporting a critical role for TAK1 in regulating TNFR1-mediated cell survival/death signaling.^24, 25, 26, 27^ Here we dissect the molecular regulatory mechanism underlying the role of TAK1 in necroptotic signaling and showed that TAK1 regulates multiple cell death checkpoints through both NF*κ*B-dependent and -independent mechanisms.

## Results

### TAK1 regulates necroptotic signaling through both NFκB-dependent and -independent mechanisms

**Figure 1:**
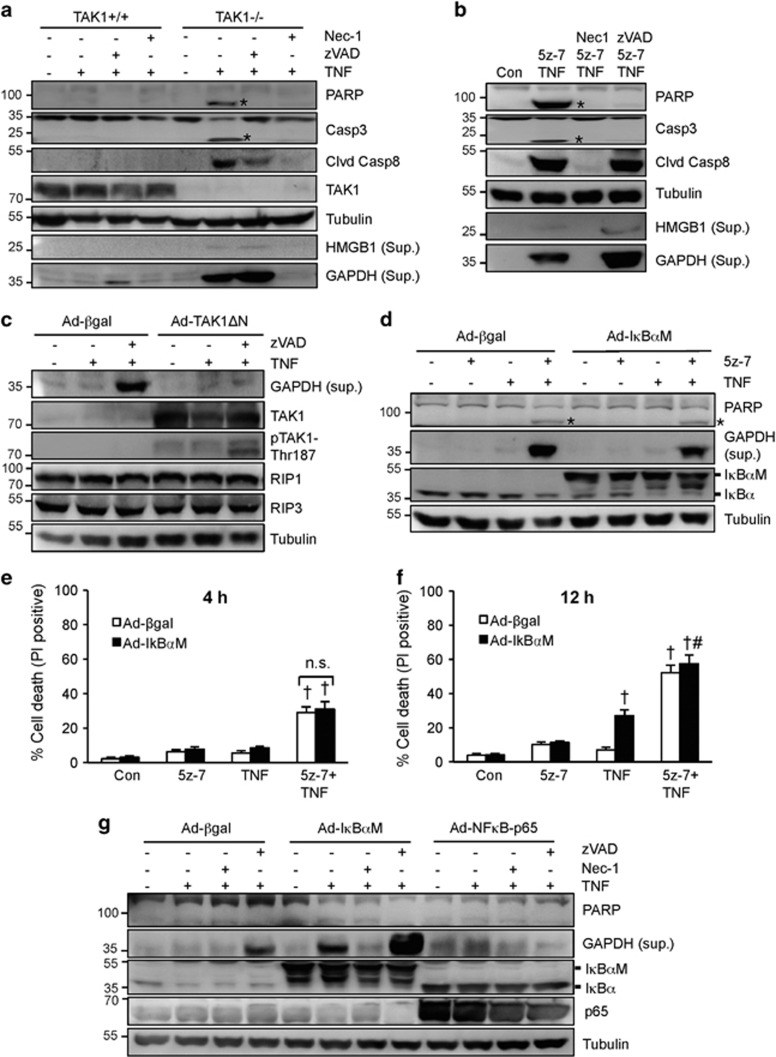
TAK1 regulates death signaling through both NF*κ*B-dependent and -independent mechanisms. (**a**) Western blots for the indicated proteins in TAK1+/+ or TAK1-/-, MEFs stimulated with 10 ng/ml TNFα or vehicle control for 4 h, with or without Nec-1 (necrostatin-1; RIP1 inhibitor) or zVAD (zVAD-fmk; pan-caspase inhibitor). (**b**) Western blots for the indicated proteins in wild-type MEFs stimulated with 10 ng/ml TNFα or vehicle control for 4 h, with or without 5z-7-oxozeaenol (5z-7; TAK1 inhibitor), Nec-1, or zVAD. (**c**) Western blots for the indicated proteins from H9c2 myocytes infected with *β*-gal or TAK1-ΔN (active mutant) adenoviruses for 24 h, followed by stimulation with vehicle control, TNF*α*, or zVAD plus TNFα for 12 h. (**d**) Western blots for the indicated proteins from H9c2 myocytes infected with *β*-gal or I*κ*B*α* mutant (I*κ*B*α*M) adenoviruses for 24 h, followed by stimulation with vehicle control, TNF*α*, 5z-7 for 4 h. (**e**) Quantification of cell death in cells treated as in **d** for 4 h. †*P*<0.05 *versus* Control. (**f**) Quantification of cell death in cells treated as in **d** for 12 h. †*P*<0.05 *versus* Control; #*P*<0.05 *versus* Ad-I*κ*BαM TNF. (**g**) Western blots for the indicated proteins from H9c2 myocytes infected with Adβ-gal, Ad-I*κ*B*α*M, or AdNF*κ*B-p65 for 24 h, followed by stimulation with vehicle control or TNFα for 12 h, in the presence or absence of zVAD or Nec-1. *indicates cleaved proteins

It has been controversial as to whether TAK1 regulates the cellular sensitivity to TNFα-induced apoptosis, necroptosis, or both.^16, 21, 23, 25^ We observed that ablation of TAK1 in mouse embryonic fibroblasts (MEFs) promoted the cleavage of PARP, caspase 8, and caspase 3 upon TNF*α* stimulation (Figure 1a). High mobility group box 1 (HMGB1), a biomarker for necroptosis,^28^ was also detected from the culture supernatant upon TNFα stimulation (Figure 1a). Intriguingly, glyceraldehyde 3-phosphate dehydrogenase (GAPDH), a cytoplasmic protein, was more readily detectable from the supernatant compared with HMGB1, indicating that the release of GAPDH may serve as a new biomarker for plasma membrane disruption/leakage (Figure 1a). Both caspase cleavage and HMGB1/GAPDH release were efficiently blocked by the RIP1 kinase inhibitor necrostatin-1 (Nec-1). Although the pan-caspase inhibitor zVAD-FMK (zVAD) blocked PARP and caspase 3 cleavage, it promoted HMGB1 and GAPDH release (Figure 1a). Similar effect was observed using a specific TAK1 inhibitor, 5z-7-oxozeanol^29^ (5z-7, Figure 1b). Of note, addition of 5z-7 did not further increase TNFα-induced cell death in TAK1-/-, MEFs, confirming the specificity of this TAK1 inhibitor (Supplementary Figure S1). Together, these data suggest that TAK1 inhibition promotes both apoptotic and necroptotic signaling.

Next, we sought to examine if forced activation of TAK1 is sufficient to inhibit necroptotic signaling. H9c2 myocytes display high transduction efficiency with adenoviral vectors and they behave similarly to wild-type MEFs in TNF*α*-induced cell death response. H9c2 cells were infected with an adenovirus encoding the constitutively active mutant of TAK1 (Ad-TAK1ΔN) or *β*-galactosidase adenovirus (Ad-*β*gal) as a control, followed by stimulation with TNFα in the presence or absence of zVAD. zVAD plus TNFα induced GAPDH release in Ad-*β*gal-infected H9c2 cells, indicating the induction of necroptosis (Figure 1c). This effect was largely blocked by TAK1ΔN overexpression, suggesting that TAK1 activation is sufficient to inhibit necroptotic signaling (Figure 1c). TAK1ΔN overexpression was associated with auto-phosphorylation on Thr187, indicating kinase activation.^30^ Of note, Ad-TAK1ΔN alone had no effect on RIP1/RIP3 expression or GAPDH release (Figure 1c).

TAK1 has been implicated as a critical regulator of TNF*α*-induced activation of NF*κ*B, a transcription factor that drives the expression of pro-survival genes. Ablation of TAK1 blocked TNF*α*-induced transient degradation of I*κ*B*α*, and inhibition of TAK1 with 5z-7 also abrogated TNF*α*-induced NF*κ*B luciferase activity (Supplementary Figure S2). To evaluate the contribution of NF*κ*B pathway to TNF*α*-induced necroptosis, H9c2 myocytes were infected with an adenovirus encoding the non-degradable I*κ*B*α* mutant (I*κ*B*α*-S32/36A; Ad-I*κ*B*α*M) that completely blocked NF*κ*B activity (Supplementary Figure S2), followed by stimulation with TNF*α* in the presence or absence of 5z-7 for 4 h. In the absence of TAK1 inhibition, abrogation of the NF*κ*B failed to induce PARP cleavage or necroptotisis following TNF*α* stimulation for 4 h (Figures 1d and e). In addition, PARP cleavage, GAPDH release, or necroptotic cell death induced by 5z-7 plus TNFα was not altered by inhibition of the NF*κ*B pathway (Figures 1d and e). These data suggest that NF*κ*B does not contribute to the acute phase of necroptosis triggered by TAK1 inhibition. Prolonged stimulation of TNFα alone for 12 h induced cell death in Ad-I*κ*BαM-infected H9c2 cells, and addition of 5z-7 further increased TNFα-induced cell death (Figure 1f). Moreover, prolonged TNF*α* stimulation promoted GAPDH release in Ad-I*κ*B*α*M-infected cells, which was blocked by the RIP1 inhibitor Nec-1 (Figure 1g). Inhibition of NF*κ*B further increased GAPDH release induced by zVAD plus TNF*α* (Figure 1g). On the other hand, overexpression of NF*κ*B-p65 in H9c2 cells, which induces robust NF*κ*B activation,^31^ blocked GAPDH release induced by zVAD plus TNF*α* (Figure 1g). These data suggest that inhibition of NF*κ*B may contribute to the delayed phase of necroptosis induced by prolonged TNF*α* stimulation.

Importantly, overexpression of NF*κ*B-p65 largely inhibited necroptotic cell death induced by 5z-7 plus TNF*α* at 4 and 12 h (Supplementary Figure S3). GAPDH release induced by 5z-7 plus TNF*α* with or without zVAD was also abrogated (Supplementary Figure S3). Therefore, our data reveal a novel anti-necroptotic role for NF*κ*B, in addition to its known anti-apoptotic function. Taken together, these data suggest that TAK1 regulates necroptotic signaling through an early NF*κ*B-independent and a delayed NF*κ*B-dependent mechanism.

### Inhibition of TAK1 triggers two caspase 8 activation pathways through the induction of RIP1-FADD-caspase 8 complex as well as FLIP degradation

**Figure 2:**
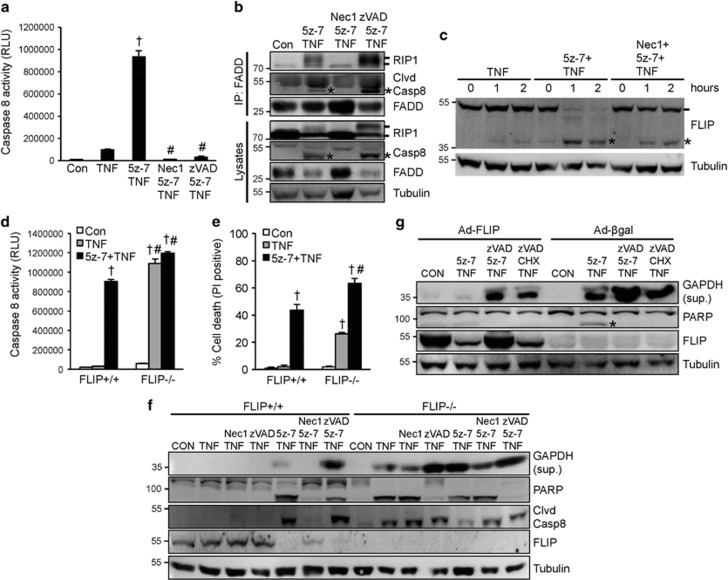
Inhibition of TAK1 triggers two caspase 8 activation pathways through induction of the RIP1-FADD-caspase 8 complex as well as FLIP degradation. (**a**) Caspase 8 activity in wild-type MEFs treated as indicated for 4 h. ^†^*P*<0.05 *versus* Control; ^#^*P*<0.05 *versus* 5z-7 plus TNF. (**b**) Western blots for the indicated proteins following IP with an anti-FADD antibody from extracts of MEFs treated as indicated for 1 h. * indicates cleaved caspase 8. (**c**) Western blots for FLIP and α-tubulin from extracts of MEFs treated as indicated. * indicates cleaved FLIP. (**d**) Caspase 8 activity in FLIP+/+ and FLIP-/-, MEFs treated as indicated for 2 h. ^†^*P*<0.05 *versus* Con; ^#^*P*<0.05 *versus* FLIP+/+. (**e**) Cell death assessed by PI staining of cells treated as in **d**. ^†^*P*<0.05 *versus* Con; ^#^*P*<0.05 *versus* FLIP+/+. (**f**) Western blots for the indicated proteins from FLIP+/+ and FLIP-/-, MEFs treated as indicated for 4 h. (**g**) Western blots for the indicated proteins from H9c2 myocytes infected with *β*-gal or FLIP adenoviruses for 24 h, then treated as indicated for 4 h. CHX, cycloheximide

**Figure 3:**
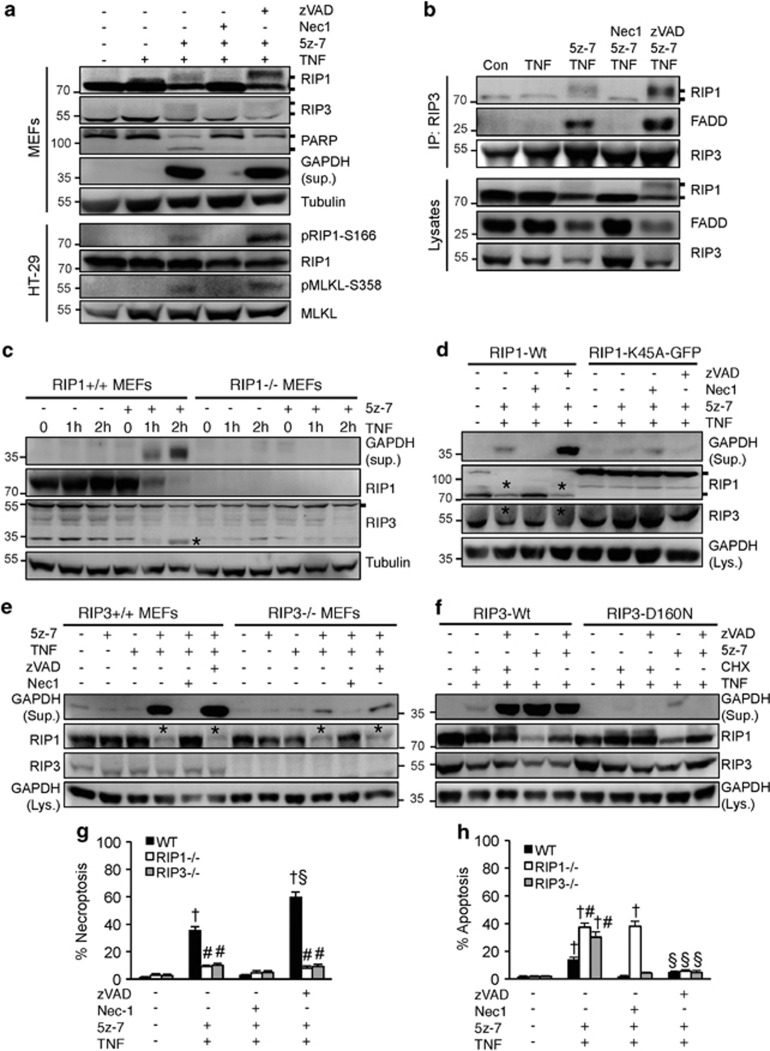
Inhibition of TAK1 promotes RIP1 phosphorylation/activation and the RIP1–RIP3-FADD necroptotic complex formation. (**a**) Western blots for the indicated proteins from MEFs or HT-29 cells treated as indicated for 4 h. (**b**) Western blots for the indicated proteins following IP with an anti-RIP3 antibody from extracts of MEFs treated as indicated for 1 h. (**c**) Western blots with the indicated antibodies from cellular extracts of RIP1+/+ and RIP1-/-, MEFs treated with TNF*α* for 0, 1, and 2 h in the presence or absence of 5z-7. (**d**) Western blots with the indicated antibodies from cellular extracts of RIP1-/-, MEFs reconstituted with wild-type (Wt) or K45A mutant of RIP1, then treated as indicated for 4 h. (**e**) Western blots with the indicated antibodies from cellular extracts of RIP3+/+ and RIP3-/-, MEFs treated as indicated for 4 h. (**f**) Western blots with the indicated antibodies from cellular extracts of RIP3-/-, MEFs reconstituted with wild-type (Wt) or the D160N mutant of RIP3, then treated as indicated for 4 h. (**g**, **h**) Necroptosis (PI positive without chromatin condensation) and apoptosis (PI negative with chromatin condensation) in wild-type, RIP1-/-, and RIP3-/-, MEFs treated as indicated for 4 h. ^†^*P*<0.05 *versus* Control; ^#^*P*<0.05 *versus* WT; ^§^*P*<0.05 *versus* 5z-7 plus TNF

Consisting with the caspase cleavage results (Figure 1a, b), TNF*α* plus 5z-7, but not TNF*α* alone, induced a rapid activation of caspase 8, which was blocked by co-treatment with Nec-1 or zVAD (Figure 2a). These data indicate that TAK1 functions to inhibit caspase activation, in addition to its anti-necroptotic effect. Wang *et al.*^9^ identified a RIP1-depedent caspase 8 activation pathway in the setting of cIAPs depletion/inhibition, by inducing the caspase 8-activating complex consisting of RIP1, FADD, and caspase 8. We hypothesize that inhibition of TAK1 may promote caspase 8 activation through a similar mechanism. Indeed, both RIP1 and caspase 8 were detected in the FADD immunoprecipitates upon simulation with TNF*α* plus 5z-7 (Figure 2b). The RIP1-FADD-caspase 8 interaction was blocked by Nec-1, but further enhanced by zVAD, indicating that RIP1 kinase activity is required for the complex formation (Figure 2b). Of note, an upshift of RIP1 was detected upon stimulation with 5z-7 plus TNF*α*, indicating RIP1 auto-phosphorylation and activation (also see Figure 3a below). Cleaved caspase 8 was also detected in the FADD immunoprecipitates following 5z-7 plus TNF*α*, suggesting the induction of an active complex II (Figure 2b). Consistent with a previous report by Biton *et al.*,^32^ zVAD failed to block the cleavage of caspase 8 into the p43 fragment, thus cleaved caspase 8 was also detectable in the FADD immunoprecipitates upon stimulation with zVAD, 5z-7, and TNF*α*.

As caspase 8 is tightly regulated by FLIP, we examined the effect of TAK1 inhibition on FLIP. Strikingly, 5z-7 plus TNFα, but not TNF*α* alone, induced a rapid cleavage/degradation of FLIP (Figure 2c). Addition of Nec-1 largely reversed this effect (Figure 2c). This result suggests that TAK1 functions to stabilize FLIP from cleavage/degradation through a RIP1-dependent mechanism. We further assessed the role of FLIP in caspase activation and necroptotic signaling using FLIP+/+ and FLIP-/- MEFs. As expected, TNFα alone greatly increased caspase 8 activity in FLIP-/- MEFs, whereas TNFα induced caspase 8 activity in FLIP+/+ MEFs only in the presence of 5z-7 (Figure 2d). Addition of 5z-7 did not further increase TNFα-induced caspase 8 activity in FLIP-/- MEFs, indicating maximal caspase 8 activation (Figure 2d). However, 5z-7 plus TNFα induced a greater level of cell death compared with TNFα alone in FLIP-/- cells (Figure 2e), suggesting that TAK1 inhibition promotes cell death through an additional, FLIP-independent mechanism. Similarly, TNFα also induced GAPDH release in FLIP-/-, MEFs, which was further enhanced with the addition of 5z-7 (Figure 2f). Intriguingly, in contrast to its effect in FLIP+/+ MEFs, Nec-1 only partially blocked GAPDH release induced by TNFα alone or 5z-7 plus TNFα in FLIP-/-, MEFs, possibly owing to the induction of a RIP1-independent cell death under these conditions (Figure 2f). Moreover, the pan-caspase inhibitor zVAD inhibited PARP cleavage but promoted GAPDH release in FLIP-/-, MEFs, indicating a switch from apoptotic to necroptotic cell death (Figure 2f). Given that TAK1 inhibition depletes endogenous FLIP, we test if restoration of FLIP could prevent necroptosis. Indeed, overexpression of FLIP in H9c2 cells partially blocked PARP cleavage as well as GAPDH release induced by 5z-7 plus TNF*α*, revealing an anti-necroptotic role for FLIP (Figure 2g). Overexpression of FLIP also partially inhibited GAPDH release induced by zVAD/5z-7/TNF*α* or zVAD/CHX/TNF*α* (Figure 2g).

Together, our data suggest that inhibition of TAK1 promotes caspase 8 activation though two independent mechanisms: the RIP1-FADD-caspase 8 complex formation and FLIP cleavage/degradation. In addition, downregulation of FLIP also contributes to the induction of necroptosis in the setting of TAK1 inhibition when zVAD is present.

### Inhibition of TAK1 promotes RIP1 phosphorylation/activation and the RIP1-RIP3-FADD necroptotic complex formation

The kinase activity of RIP1 and RIP3 is essential for the necrosome formation and necroptotic cell death.^3, 4, 5^ Here, we assessed whether TAK1 exerts its anti-necroptotic effect through RIP1 and/or RIP3. Importantly, 5z-7 plus TNF*α*, but not TNF*α* alone, induced an upshift of RIP1 and RIP3 on SDS-PAGE, which was blocked by Nec-1, but further enhanced by zVAD (Figure 3a). This result suggests that TAK1 may regulate phosphorylation of RIP1/3. Using an anti-phospho-RIP1 (Ser166) and an anti-phospho-MLKL (Ser358) antibody, we were able to detect both RIP1 and MLKL phosphorylation in HT-29 cells following stimulation with 5z-7 plus TNF*α*, especially in the presence of zVAD (Figure 3a), confirming the induction of necroptotic signaling. Next, we examined if TAK1 inhibition could induce the formation of the RIP1-RIP3-FADD necroptotic complex. Indeed, both RIP1 and FADD were detected in the RIP3 immunoprecipitates from cells treated with 5z-7 plus TNF*α*, and the RIP1-RIP3-FADD interaction was inhibited by Nec-1, but not by zVAD (Figure 3b). Thus, TAK1 exerts its anti-necroptotic effect by preventing RIP1 phosphorylation/activation and the RIP1-RIP3-FADD complex formation.

Consistent with an essential role for RIP1 in death signaling, deletion of RIP1 completely blocked GAPDH release as well as RIP3 cleavage induced by 5z-7 plus TNF*α* (Figure 3c). To directly test if the kinase activity of RIP1 is required in this process and to exclude possible off-target effect of the RIP1 inhibitor Nec-1, MEFs stably expressing wild-type (Wt) or the kinase-dead mutant (K45A) of RIP1 were generated by re-expressing these proteins in RIP1-/- cells. 5z-7 plus TNFα induced GAPDH release and RIP3 cleavage in RIP1-Wt, but not RIP1-K45A MEFs (Figure 3d). Similarly, GAPDH release induced by 5z-7 plus TNF*α* was blocked in RIP3-/- MEFs as well as in cells expressing the kinase inactive mutant of RIP3 (D160N) (Figures 3e and f). However, RIP1 cleavage induced by 5z-7 plus TNFα was not affected in RIP3-deficient cells, suggesting that RIP3 acts downstream of RIP1 in necroptotic signaling (Figures 3e and f).

Consistent with the data above, necroptosis induced by 5z-7 plus TNF*α* was largely blocked by ablation of RIP1 or RIP3 (Figures 3g and h). Interestingly, ablation of RIP1 or RIP3 induced a moderate increase in apoptosis following stimulation with 5z-7 plus TNF*α*, which was blocked by zVAD (Figures 3g and h). Ablation of RIP3 also slightly enhanced 5z-7 plus TNF*α*-induced caspase 8 activity (Supplementary Figure S4). On the other hand, inhibition of caspases with zVAD further enhanced necroptosis in wild-type MEFs (Figures 3g and h). These data also support a cross-talk between necroptosis and apoptosis that is delicately regulated by RIP1/RIP3 and caspases.

### The adaptor protein TRADD is essential for caspase 8 activation and necrosome formation in the setting of TAK1 inhibition

**Figure 4:**
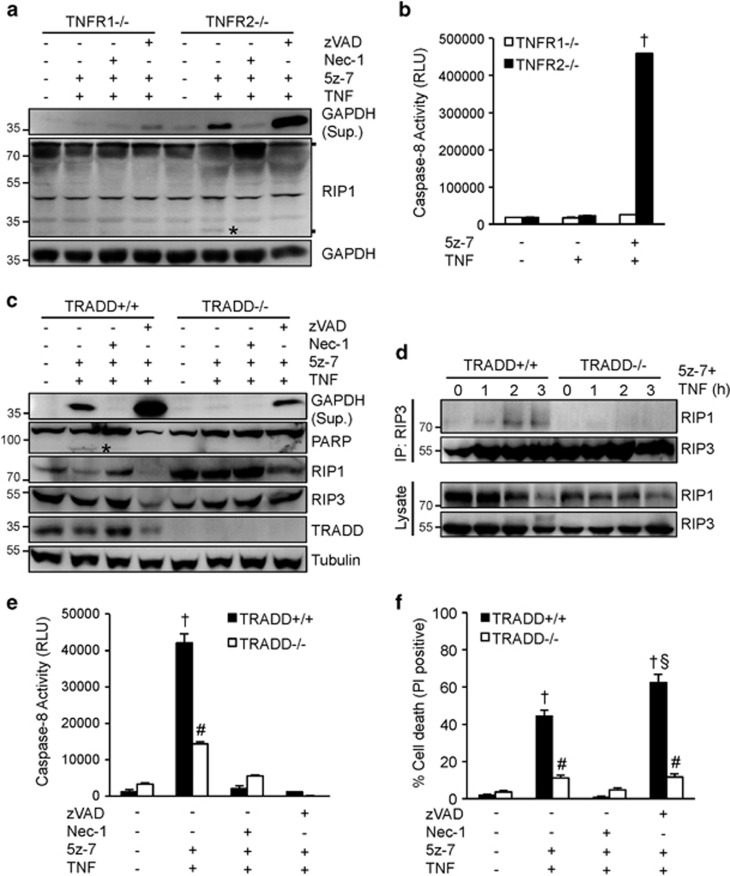
The adaptor protein TRADD is indispensible for caspase 8 activation and necrosome formation in the setting of TAK1 inhibition. (**a**) Western blots for the indicated proteins from TNFR1-/-, and TNFR2-/- MEFs treated with vehicle control or 5z-7 plus TNF*α* for 4 h, in the presence or absence of Nec-1 or zVAD. (**b**) Caspase 8 activity in TNFR1-/-, and TNFR2-/-, MEFs treated with vehicle control, TNF*α*, or 5z-7 plus TNF*α* for 2 h. †*P*<0.01 *versus* Control or TNF only. (**c**) Western blots for the indicated proteins from TRADD+/+ and TRADD-/-, MEFs treated with vehicle control or 5z-7 plus TNF*α* for 4 h, in the presence or absence of Nec-1 or zVAD. (**d**) Western blots for the indicated proteins following IP with an anti-RIP3 antibody from TRADD+/+ and TRADD-/-, MEFs treated with 5z-7 plus TNF*α* for the indicated periods of time. (**e**) Caspase 8 activity in TRADD+/+ and TRADD-/-, MEFs treated with vehicle control or 5z-7 plus TNF*α* for 2 h, with or without Nec-1 or zVAD. †*P*<0.01 *versus* Con; ^#^*P*<0.05 *versus* corresponding TRADD+/+. (**f**) Cell death assessed by PI staining of TRADD+/+ and TRADD-/-, MEFs treated as in **e** for 4 h. ^†^*P*<0.01 *versus* Con; ^#^*P*<0.05 *versus* corresponding TRADD+/+ ^§^*P*<0.05 *versus* TRADD+/+ 5z-7 plus TNF. * indicates cleaved proteins

To gain further mechanistic insights, we carefully examined the role of several key components of TNFR1 signaling pathway in necroptotic cell death triggered by TAK1 inhibition. Both TNFR1 and TNRF2 have been shown to mediate necroptotic cell death.^33^ Here we determined that ablation of TNFR1, but not TNFR2, blocked GAPDH release and caspase 8 activation induced by 5z-7 plus TNF*α* (Figures 4a and b). These data indicate that TAK1 regulates cellular sensitivity to TNFα-induced necroptotic signaling and caspase activation mainly through TNFR1. Following TNFR1 ligation, the adapter protein TRADD is rapidly recruited to the death domain of TNFR1, which is critical for the formation of downstream signaling complexes.^34^ However, the role of TRADD in necroptotic signaling has not been investigated. Here we showed that deletion TRADD largely blocked PARP cleavage, caspase 8 activation, GAPDH release, and cell death induced by 5z-7 plus TNFα, indicating a critical role for TRADD in regulating both apoptotic and necroptotic signaling (Figures 4c and f). Importantly, deletion of TRADD also prevented the RIP1/RIP3 necrosome formation induced by 5z-7 plus TNF*α* (Figure 4d). Therefore, these data revealed an essential role for the adaptor protein TRADD in regulating caspase 8 activation and necrosome formation in the setting of TAK1 inhibition.

### Ablation of the RIP1 deubiquitinase CYLD prevents necroptotic signaling triggered by TAK1 inhibition

**Figure 5:**
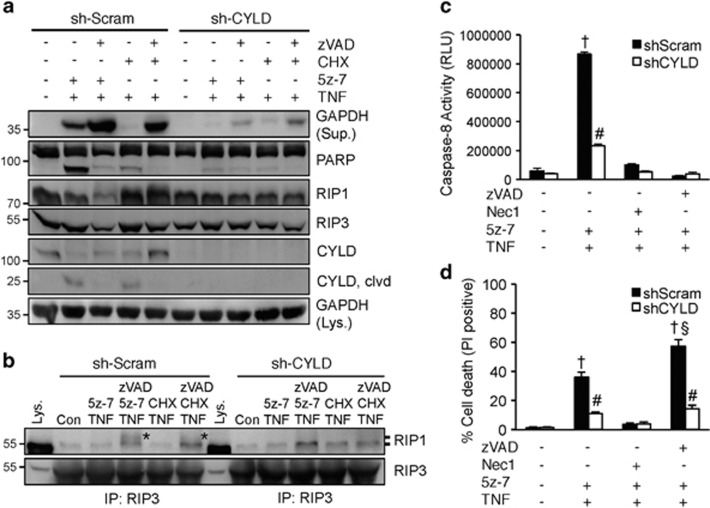
Ablation of the RIP1 deubiquitinase CYLD prevents death signaling triggered by TAK1 inhibition. (**a**) Western blots for the indicated proteins from MEFs stably expressing CYLD shRNA (shCYLD) or a scrambled sequence (shScram) by lentiviral vectors, treated with 5z-7 plus TNFα or CHX plus TNF*α*, with or without zVAD for 4 h. (**b**) Western blots for RIP1 and RIP3 after IP with an anti-RIP3 antibody from extracts of shScram or shCYLD MEFs treated as indicated for 1 h. *indicates mobility shift. (**c**) Caspase 8 activity in shScram and shCYLD MEFs treated with vehicle control or 5z-7 plus TNFα in the presence or absence of Nec-1 or zVAD for 2 h. ^†^*P*<0.01 *versus* Con; #*P*<0.05 *versus* corresponding shScram. (**d**) Cell death assessed by PI staining of cells treated as in **c** for 4 h. ^†^*P*<0.01 *versus* Con; #*P*<0.05 *versus* corresponding shScram; ^§^*P*<0.05 *versus* 5z-7 plus TNF

Next, we tested whether the RIP1 deubiquitinase CYLD is required the TNFα-induced necroptosis in the setting of TAK1 inhibition. Remarkably, silencing of CYLD with a shRNA lentiviral vector largely blocked PARP cleavage and GAPDH release induced by 5z-7 plus TNF*α* (Figure 5a). Of note, GAPDH release induced by zVAD/5z-7/TNFα or zVAD/CHX/TNFα was also largely inhibited in CYLD-deficient cells (Figure 5a). In addition, 5z-7 plus TNFα also induced CYLD cleavage, which was reversed by zVAD, although it is unclear how CLYD cleavage affects necroptotic signaling. Further, silencing CYLD blocked the RIP1/RIP3 necrosome formation induced by zVAD/5z-7/TNFα or zVAD/CHX/TNFα. RIP1 phosphorylation, as indicated by the mobility shift, was also attenuated (Figure 5b). Consistent with the data above, silencing CYLD largely abolished caspase 8 activation and necroptotic cell death induced by 5z-7 plus TNF*α* (Figures 5c and d). These data suggest CYLD is critically involved in TAK1-mediated regulation of apoptotic and necroptotic signaling.

### Blockade of the ubiquitin-proteasome pathway attenuates protein degradation and necroptotic signaling triggered by TAK1 inhibition

**Figure 6:**
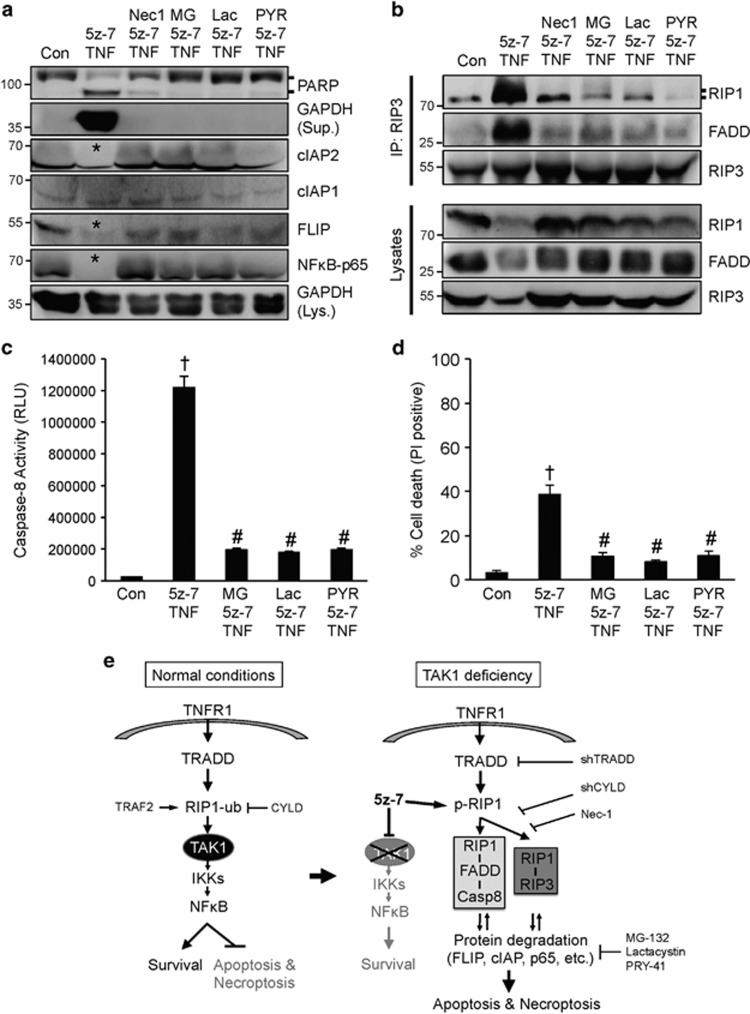
Blockade of the ubiquitin-proteasome pathway attenuates protein degradation and necroptotic signaling triggered by TAK1 inhibition. (**a**) Western blots for the indicated proteins from extracts of wild-type MEF treated with vehicle control or 5z-7 plus TNFα for 4 h, in the presence of Nec-1, MG-132 (MG), lactacystin (Lac), or PYR-41 (PYR). * Indicates protein degradation. (**b**) Western blots for the indicated proteins after IP with an anti-RIP3 antibody from extracts of cells treated as in **a** for 1 h. (**c**) Caspase 8 activity in cells treated as in **a** for 2 h. ^†^*P*<0.01 *versus* Con; ^#^*P*<0.05 *versus* 5z-7 plus TNF. (**d**) Cell death assessed by PI staining in cells treated as in **a** for 4 h. ^†^*P*<0.01 *versus* Con; ^#^*P*<0.05 *versus* 5z-7 plus TNF. (**e**) Proposed model: TAK1 functions as nodal regulator of apoptosis and necroptosis by regulating multiple cell death checkpoints

Importantly, TAK1 inhibition triggered the degradation of several signaling molecules that regulate apoptosis and necroptosis, including cIAP2, FLIP, and NF*κ*B-p65 (Figure 6a). This prompted us to assess the potential role of ubiquitin-proteasome pathway in regulating necroptosis. In addition to clearing damaged and misfolded proteins, the ubiquitin-proteasome pathway also regulates the availability of key signaling proteins in various cellular processes such as cell growth and proliferation, cell cycle regulation, and gene expression. Using specific proteasome inhibitors MG-132, lactacystin, or the ubiquitin-activating enzyme E1 inhibitor PYR-41, we showed that blocking the ubiquitin-proteasome pathway effectively inhibited PARP cleavage and GAPDH release induced by 5z-7 plus TNFα, an effect comparable to Nec-1 (Figure 6a). More importantly, the degradation of cIAP2, FLIP, and NF*κ*B-p65 was also largely abolished (Figure 6a). We further examined whether inhibition of proteasome pathway affects the necrosome formation. Indeed, similar to Nec-1, pretreatment with MG-132, lactacystin, or PYR-41 also blocked the RIP1–RIP3-FADD necroptotic complex formation induced by 5z-7 plus TNF*α* (Figure 6b). Pretreatment with MG-132, lactacystin, or PYR-41 also attenuated caspase 8 activity and necroptotic cell death induced by 5z-7 plus TNF*α* (Figures 6c and d). Taken together, these data suggest that the ubiquitin-proteasome pathway regulates death signaling by controlling the availability of key pro-survival signaling proteins FLIP, cIAP2, and NF*κ*B-p65.

## Discussion

Our results demonstrated that TAK1 functions as a nodal regulator of the TNFR1-mediated cell survival/death signaling through regulation of multiple cell death checkpoints. Here we showed that inhibition of TAK1 inactivates NF*κ*B, a cell death checkpoint for apoptosis and necroptosis (Figure 6e). TAK also regulates cell death through an acute NF*κ*B-independent mechanism involving the induction of two cell death complexes: the caspase 8-activating complex (RIP1-FADD-capse 8) and the necroptotic cell death complex (RIP1–RIP3-FADD) (Figure 6e). Inhibition of TAK1 promoted phosphorylation and activation of RIP1, which is essential for the formation of both cell death complexes. Ablation of the RIP1 deubiquitinase CYLD largely blocked apoptotic and necroptotic cell death induced by TAK1 inhibition. We also identified an indispensable role for the adaptor protein TRADD in TNF*α*-induced necroptotic signaling. Finally, we identified the ubiquitin-proteasome pathway as a novel necroptotic regulatory mechanism by controlling the availability of key pro-survival signaling proteins, including FLIP, cIAPs, and NF*κ*B (Figure 6e).

Although the anti-apoptotic role of the NF*κ*B has been well established, its role in regulating necroptosis remains elusive.^25^ Here we provide evidence that the acute phase of TNF*α*-induced necroptosis triggered by TAK1 inhibition is NF*κ*B-independent but the late-onset necroptosis is NF*κ*B-dependent. Moreover, activation of NF*κ*B by overexpressing p65 blocked both apoptotic and necroptotic signaling induced by 5z-7 plus TNF*α*. Similarly, overexpression of IKK2-EE, an NF*κ*B activator, was shown to inhibit TRAIL-induced cell death in TAK1-/-, MEFs.^35^ The anti-necroptotic effect of NF*κ*B was likely due to the upregulation of pro-survival genes such as FLIP and cIAPs. Indeed, overexpression of FLIP partially abrogated necroptotic cell death induced by 5z-7 plus TNF*α*. Therefore, our data support a model that TAK1 prevents apoptotic and necroptotic cell death through both NF*κ*B-independent and NF*κ*B-dependent checkpoints.

We showed that RIP1 kinase activity is essential for the induction of cell death complexes and apoptotic/necroptotic cell death in TAK1-deficient cells. We speculate that TAK1 inhibition may promote RIP1 phosphorylation/ activation through degradation of cIAPs. Indeed, depletion of cIAPs has been shown to induce RIP1 phosphorylation/activation in L929 cells.^22^ Moreover, our recent studies showed that TAK1 prevents cell death complex formation by interacting and stabilizing RIP1 in the complex I.^24^ When TAK1 is inhibited or depleted, RIP1 switches its binding partner by dissociating from TAK1 and associating with FADD/caspase 8 or RIP3 to form the caspase 8-activating complex or the RIP1–RIP3 necrosome. In line with these results, forced activation of TAK1 blocked cell death complex formation and necroptotic cell death, further suggesting that TAK1 exerts its anti-necroptotic effect by preventing RIP1 activation.

Although TRADD has been shown to be indispensable for TNF*α*-induced apoptosis,^34, 36^ its role in necroptotic signaling remains unclear.^37, 38^ Our data uncovered an essential role of TRADD in necroptotic signaling in the setting of TAK1 inhibition and established TRADD as an upstream regulator of both apoptotic and necroptotic signaling. Whether CYLD is required for TNFα-induced necroptosis has been controversial.^8, 15, 22^ Here we showed that ablation of CYLD largely blocked caspase activation as well as the RIP1–RIP3 necrosome formation induced by TAK1 inhibition. We speculate that the deubiquinating activity of CYLD is required for RIP1 kinase activation and subsequent necrosome formation in the setting of TAK1 inhibition. In addition, CYLD has also been shown to function as a deubiquitinating enzyme for TAK1 and negatively regulates its activity.^39^ Thus, repression of CYLD may prevent cell death at the level of both TAK1 and RIP1.

Finally, we identified the ubiquitin-proteasome pathway as a previously unidentified regulatory mechanism of necroptosis. Inhibition of TAK1 promoted destabilization and degradation of key proteins of the necroptotic signaling pathway, including FLIP, cIAPs, NF*κ*B-p65, and possibly other signaling proteins. Importantly, inhibition of the ubiquitin-proteasome pathway attenuated protein degradation, caspase activation, necrosome formation, as well as necroptotic cell death, thus revealing a critical role for the ubiquitin-proteasome pathway in regulating necroptosis. Further study is needed to determine how the ubiquitin-proteasome pathway is regulated in necroptosis and how this pathway can be specifically targeted in pathological conditions. In summary, we identified new regulatory mechanisms underlying the critical role of TAK1 in necroptotic signaling through regulation of multiple cell death checkpoints. Targeting key components of the necroptotic pathway (e.g., TRADD and CYLD) and the ubiquitin-proteasome pathway may represent a valid therapeutic strategy for pathological conditions driven by necroptosis.

## Materials and Methods

### Experimental reagents

5z-7-oxozeaenol, necrostatin-1, cycloheximide, MG-132, lactacystin, and PYR-41 were purchased from Sigma (St. Louis, MO, USA). Mouse TNF*α* was from R&D Systems (Minneapolis, MN, USA). zVAD-FMK was obtained from Abcam (Cambridge, MA, USA). Propidium iodide (PI), Hoechst 33342, and puromycin dihydrochloride were from Invitrogen. hRIP1-wt, hRIP1-K45A, hRIP3-wt, and hRIP3-K45A plasmids were obtained from Addgene (Cambridge, MA, USA).

### Cell lines

H9c2 myoblasts and HT-29 cells were obtained from American Type Culture Collection (Manassas, VA, USA). TNFR1-/- and TNFR2-/- MEFs were kindly provided by David Vaux (Walter and Eliza Hall Institute Biotechnology Centre, Australia). TAK1+/+ and TAK1-/- MEFs were a gift from Shizuo Akira (Osaka University, Japan). FLIP+/+ and FLIP-/- MEFs were provided by John Harlan (University of Washington University). TRAF2+/+ and TRAF2-/- MEFs were from TAK Mak (University Health Network, Canada). TRADD+/+ and TRADD-/- MEFs were from Manolis Pasparakis (University of Cologne, Germany). RIP1-/- and RIP3-/- MEFs were obtained from Michelle Kelliher (Universty of Massachusetts), Zheng-gang Liu (National Institutes of Health), and Francis Chan (University of Massachusetts). RIP1-/- MEFs were reconstituted with wild-type or K45A mutant of RIP1 using a retroviral vector. Similarly, RIP3-/- MEFs were reconstituted with wild-type or D160N mutant of RIP3. Stably transduced cells were selected with 8 *μ*g/ml puromycin (Invitrogen). Cells were grown in Dulbecco's modified Eagle's medium supplemented with 10% fetal bovine serum, 100 *U*/ml penicillin, 100 *μ*g/ml streptomycin, and 2 mM glutamine.

### Cell death assays

Cell death was measured using a Cell Meter Apoptotic and Necrotic Detection kit (ATT Bioquest, Sunnyvale, CA, USA) as previously described.^24^ In brief, cells were incubated at 37 °C for 30 min with Apopxin Green for detection of phosphatidylserine on cell surface, PI or 7-ADD for labeling the nucleus of cells with membrane rupture, and CytoCalcein for labeling live cell cytoplasm. Cell death was then analyzed with an EVOS FL digital fluorescence microscope (AMG) or a FACSCalibur flow cytometer (BD Biosciences, San Jose, CA, USA). Cells with chromatin condensation were visualized by Hoechst 33342 (Invitrogen, Waltham, MA, USA) staining. Cell viability was also assessed using the Muse Count & Viability assay kit (Millipore, Billerica, MA, USA). In brief, cells were trypsinized, washed, and incubated with the Muse Count & Viability reagent, and cell viability was quantified on a Muse cell analyzer (Millipore).

### Adenoviral infection

Ad-*β*gal, Ad-TAK1-ΔN, AdNF*κ*B-p65, and Ad-I*κ*BαM have been described previously.^30, 31^ AdNF*κ*B luciferase reporter and Ad-FLIP were obtained from Vector Biolabs (Philadelphia, PA, USA). Adenoviral infections were performed as described previously at a multiplicity of infection of 10 to 50 plaque forming units per ml.^30, 40^ Cells were harvested 24 h after infection followed by western blot analysis, luciferase assay, or cell death assays.

### Luciferase assays

Luciferase reporter assays was performed as described.^30, 40^ In brief, cells expressing the NF*κ*B luciferase reporter were washed in PBS and then resuspended in lysis buffer (100 mM KH_2_PO4, pH 7.8, 0.5% Nonidet P-40, and 1 mM DTT). Cellular lysates were centrifuged at 3 000 *g* for 10 min at 4 °C and supernatants were assayed in the luciferase assay buffer (100 mM Tris-HCl, pH 7.8, 10 mM Magnesium acetate, 1 mM EDTA, 1 mM DTT, 2 mM ATP, and 1 mM luciferin). Luminescence was determined with a Synergy 2 Multi-Mode Microplate Reader (BioTek, Winooski, VT, USA).

### Caspase 8 activity assay

Caspase 8 activity assay was performed using the Caspase-Glo 8 Assay kit from Promega following the manufacturer's instructions. Briefly, 100 *μ*l of Caspase-Glo 8 reagent was added to the cell culture medium in a 96-well plate. Contents of wells were gently mixed using a plate shaker at 500 rpm for 30 s. Luminescence was measured with a Synergy 2 Multi-Mode Microplate Reader (BioTek).

### shRNA-mediated knockdown

Lentiviral particles encoding shRNA sequences for specific target genes were obtained from Sigma. MEFs or H9c2 cells were seed at appropriate density in growth media containing 5 *μ*g/ml hexadimethrine bromide (Sigma), and cells were then infected by adding shRNA lentiviral particles to the culture. Stable clones expressing the shRNA were selected using 2–10 *μ*g/ml puromycin dihydrochloride. shRNA-mediated knockdown was confirmed by western blotting.

### Western blot analysis

Western blotting followed by enhanced chemiluminescence detection was performed as previously described.^24^ In some experiments, cell culture supernatants were also collected for the detection of HMGB1 or GAPDH. The following antibodies were used: Anti-TAK1 (4505), anti-phospho-TAK1 (Thr187; 4536), anti-α tubulin (3873), anti-HMGB1 (3935), anti-PARP (9532), anti-caspase 8 (4790), anti-cleaved caspase 8 (9429), anti-caspase 3 (9662), anti-RIP1 (3493), anti-CYLD (8462), anti-TRADD (3694), anti-cIAP1 (4952), and anti-FLIP (8510) were from Cell Signaling Biotechnology (Beverly, MA, USA); anti-RIP3 (sc-135171), anti-FADD (sc-6036), anti-CYLD (sc-74435), anti-I*κ*Bα (sc-847), anti-p65 (sc-372), anti-cIAP2 (sc-7944), and anti- GAPDH antibodies were from Santa Cruz Biotechnology (Santa Cruz, CA, USA); anti-RIP1 (610459) was from BD Biosciences; anti-FLIP (XA-1008) was from ProSci (Poway, CA, USA); anti-FADD (AD-I-AAM-212-E) was from Enzo Life Sciences (Farmingdale, NY, USA).

### Immunoprecipitation

Immunoprecipitation was performed as described previously.^24, 31^ Cells were lysed at 4 °C in lysis buffer (50 mM Tris-HCl (pH 7.5), 150 mM NaCl, 1 mM EDTA, 10 mM NaF, 1 mM sodium vanadate, 0.5% NP-40) containing protease and phosphatase inhibitor cocktail (Roche, Indianapolis, IN, USA). Whole cell lysates were cleared by centrifugation at 18 000 *g* for 10 min and then incubated with 2 *μ*g antibodies as indicated and protein A/G-PLUS agarose beads (Santa Cruz Biotechnologies) overnight at 4 °C. The beads were washed extensively with wash buffer (0.3% NP-40 in PBS), and the proteins were resolved on an 8-12% SDS-PAGE for subsequent western blotting.

### Statistics

All results are presented as mean±S.E.M. Data were evaluated by Student's *t*-test (for two group comparison) or one-way ANOVA with the Bonferroni's *post hoc* test (for multiple group comparison). *P*<0.05 was considered statistically significant.

## Abbreviations

TAK1: TGF*β*-activated kinase 1
TNFR1: tumor necrosis factor receptor 1
TRADD: TNF receptor-associated protein with death domain
TRAF2: TNF receptor-associated factor 2
CYLD: cylindromatosis
FLIP: FLICE-like inhibitory protein
RIP1/3: receptor-interacting protein 1 and 3
cIAP1/2: cellular inhibitor of apoptosis protein 1 and 2
FADD: Fas-associated protein with death domain
HMGB1: high mobility group box 1
GAPDH: glyceraldehyde 3-phosphate dehydrogenase
5z-7: 5z-7-oxozeanol
Nec-1: necrostatin-1
zVAD: zVAD-FMK
