# Supplementary material for: TAK1 regulates caspase 8 activation and necroptotic signaling via multiple cell death checkpoints
Source: Cell Death Dis. 2016 Sep 29;7(9):e2381–. doi: 10.1038/cddis.2016.294 (PMC5059887; doi:10.1038/cddis.2016.294)
Supplement: Supplementary Figures [file cddis2016294x1.pdf]

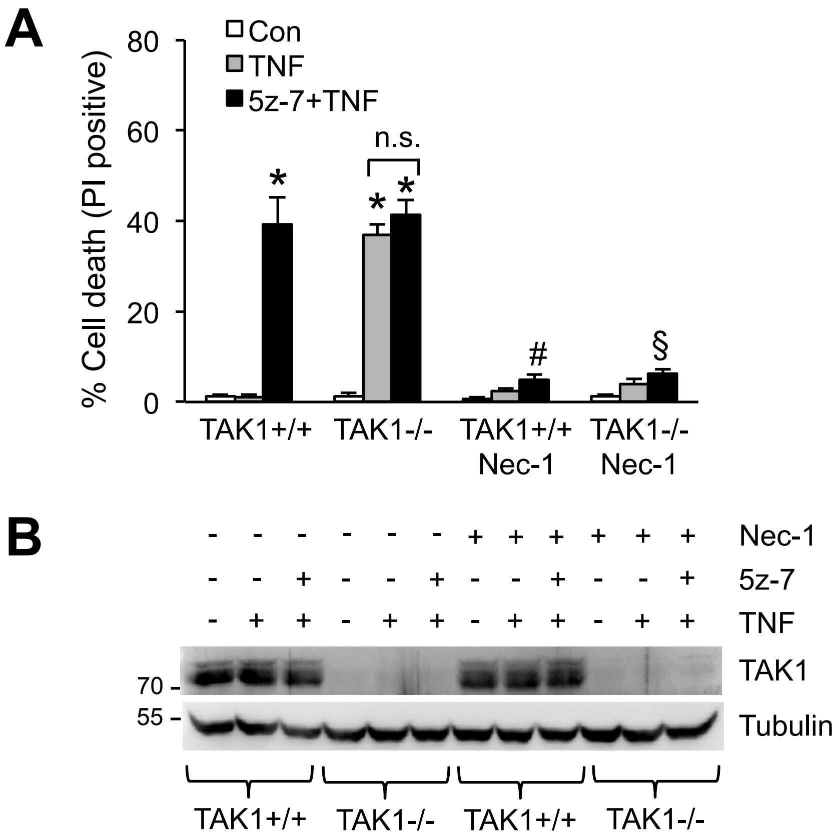

Figure S1. Inhibition of TAK1 with 5z-7 promotes TNFα-induced cell death. **A.** Cell death assessed by propidium iodide (PI) staining of TAK1+/+ and TAK1-/- MEFs treated with vehicle control, TNFα, or 5z-7 plus TNFα, with or without Nec-1. **P* < 0.05 vs Con; #*P* < 0.05 vs TAK1+/+ 5z-7+TNF; §*P* < 0.05 vs TAK1-/- 5z-7+TNF. n.s, non-significant. **B.** Western blots for the indicated proteins from TAK1+/+ and TAK1-/- MEFs.

A

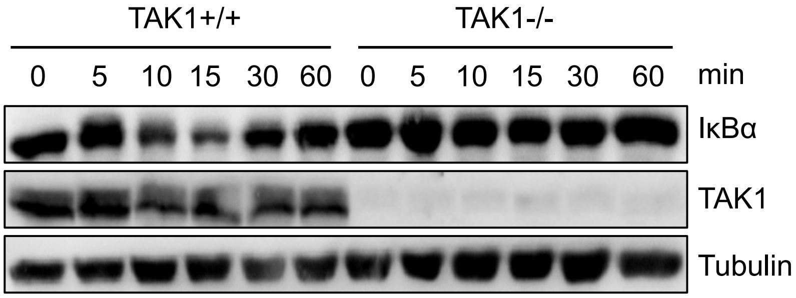

B

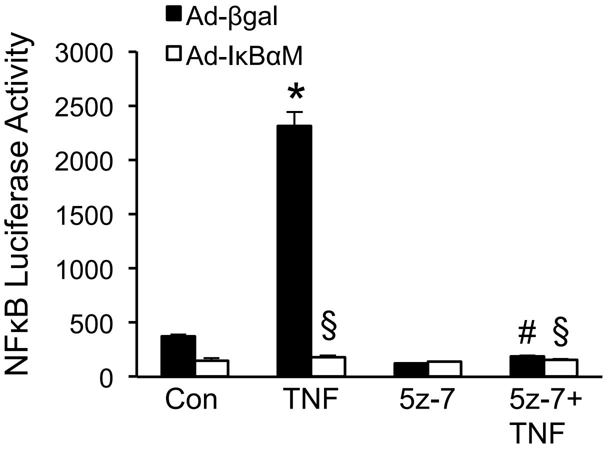

Figure S2. TAK1 is essential for TNFα-induced NFκB activation. **A.** Western blotting with the indicated antibodies from cellular extracts of TAK1+/+ and TAK1-/- MEFs stimulated with 10 ng/ml TNFα for the indicated time periods. **B.** Relative NFκB-luciferase activity from H9c2 myocytes infected with Ad-βgal or Ad-IκBαM (IκBα S32/36A mutant) along with an adenovirus encoding the NFκB-luciferase reporter for 24 h, followed by treatment with vehicle control, TNFα, 5z-7, or 5z-7 plus TNFα for 12 h. *$P < 0.01$ vs Con; #$P < 0.05$ vs TNF. §$P < 0.05$ vs Ad-βgal TNF.

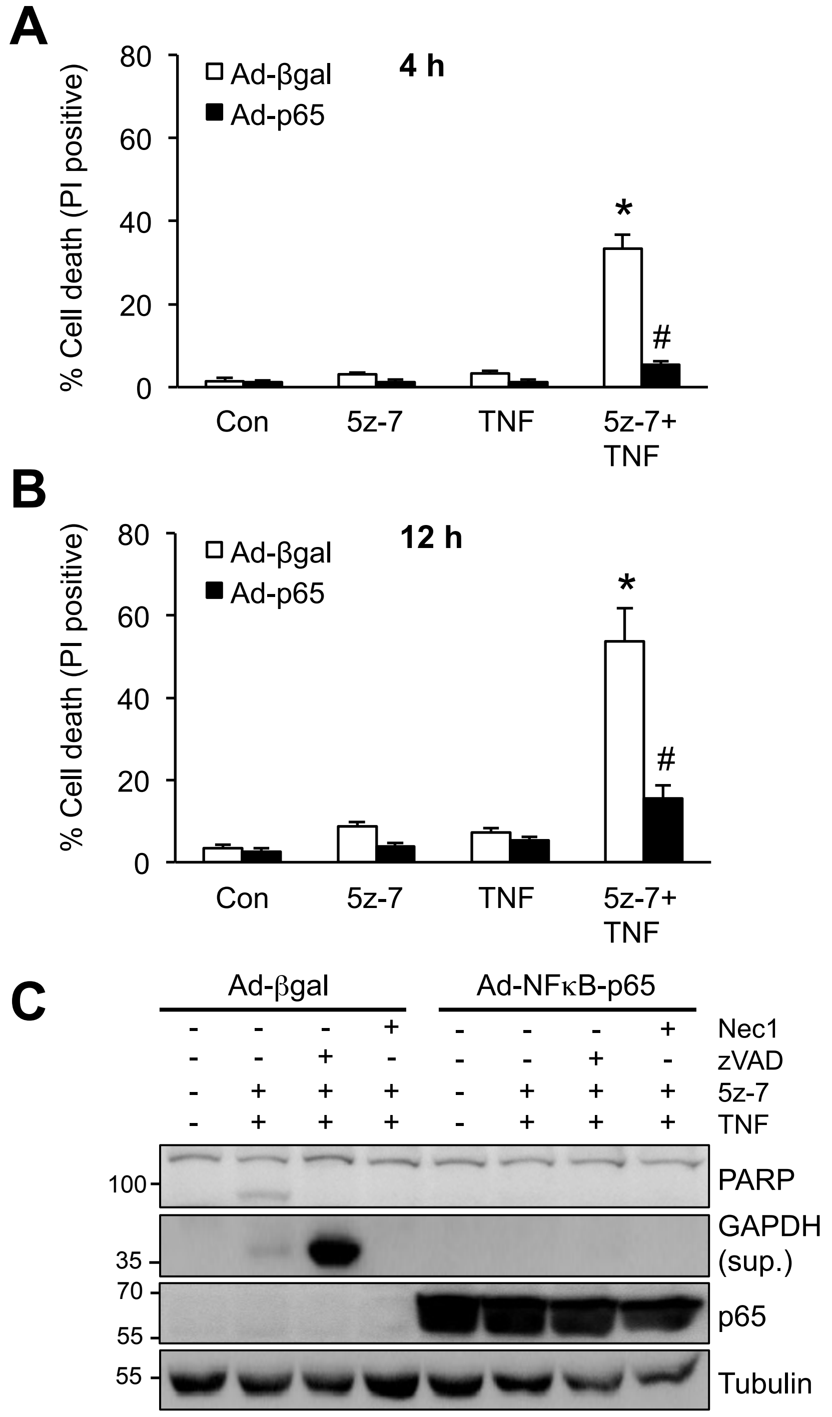

Fig S3. NFκB-p65 inhibits necroptotic cell death triggered by TAK1 inhibition. **A.** Quantification of cell death in H9c2 myocytes infected with β-gal or NFκB-p65 adenoviruses for 24 h, followed by stimulation with vehicle control, TNFα, 5z-7 for 4 h. **P* < 0.05 vs Con. #*P* < 0.05 vs Ad-βgal 5z-7+TNF. **B.** Quantification of cell death in cells treated as in A for 12 h. **P* < 0.05 vs Con; #*P* < 0.05 vs Ad-βgal 5z-7+TNF. **C.** Western blots for the indicated proteins from H9c2 myocytes infected with β-gal or NFκB-p65 adenoviruses for 24 h, followed by treatment with vehicle control or TNFα for 4 h, in the presence or absence of 5z-7 or Nec-1.

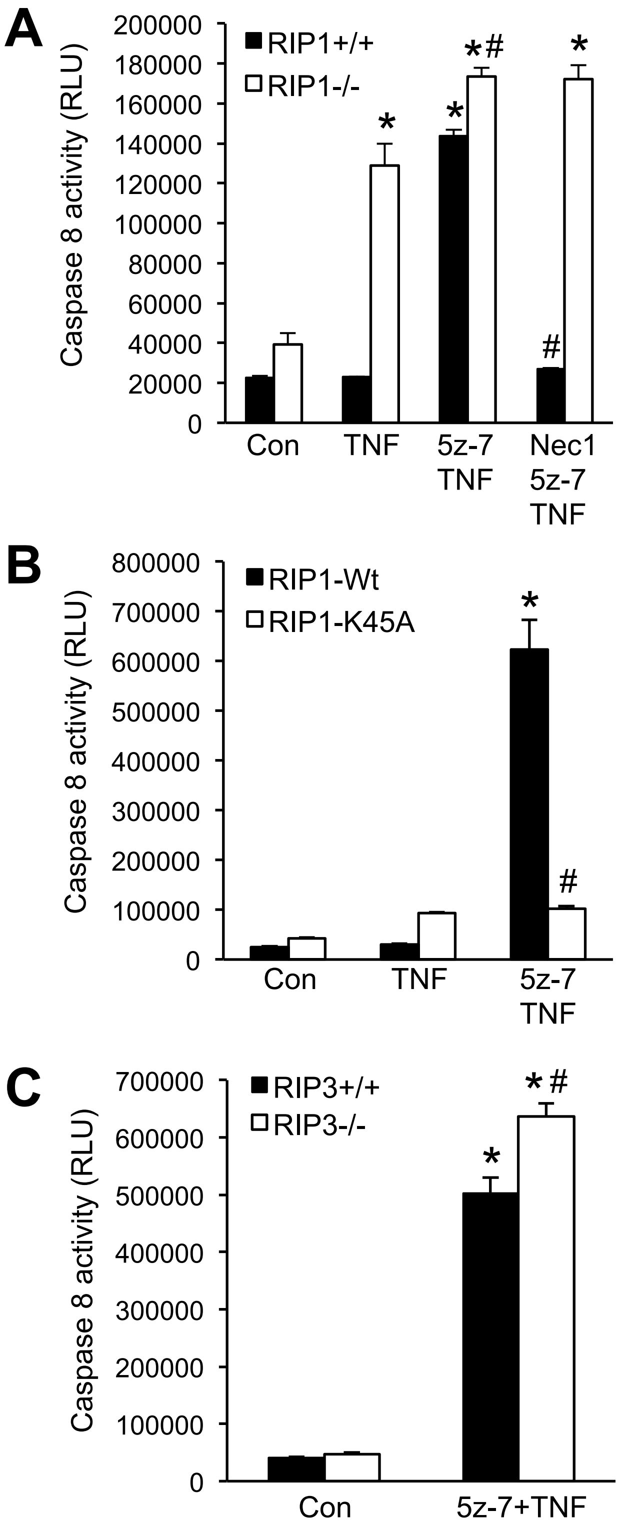

Figure S4. Role of RIP1 and RIP3 in TNFα-induced caspase 8 activity in the setting of TAK inhibition. **A.** Caspase 8 activity in RIP1+/+ and RIP1-/- MEFs treated as indicated for 4 h. **P* < 0.01 vs Con; #*P* < 0.05 vs RIP1+/+ 5z-7 plus TNF. **B.** Caspase 8 activity in RIP1-/- MEFs reconstituted with wild-type (Wt) or K45A mutant of RIP, treated as indicated for 4 h. **P* < 0.05 vs Con; #*P* < 0.05 vs RIP1-Wt. **C.** Caspase 8 activity in RIP3+/+ and RIP3-/- MEFs treated with vehicle control or 5z-7 plus TNFα for 4 h. **P* < 0.05 vs Con; #*P* < 0.05 vs RIP3+/+.
